# Development and validation of a machine learning-based risk model for metastatic disease in nmCRPC patients: a tumor marker prognostic study

**DOI:** 10.1097/JS9.0000000000002321

**Published:** 2025-03-26

**Authors:** Xudong Ni, Ziyun Wang, Xiaomeng Li, Jixinnan Sui, Weiwei Ma, Jian Pan, Dingwei Ye, Yao Zhu

**Affiliations:** a Department of Urology, Fudan University Shanghai Cancer Center, Shanghai, China; b Department of Oncology, Shanghai Medical College, Fudan University, Shanghai, China; c Shanghai Genitourinary Cancer Institute, Shanghai, China

**Keywords:** machine learning, metastasis-free survival, nonmetastatic castration-resistant prostate cancer, prognostic model, prostate cancer

## Abstract

**Background:**

Nonmetastatic castration-resistant prostate cancer (nmCRPC) is a clinical challenge due to the high progression rate to metastasis and mortality. To date, no prognostic model has been developed to predict the metastatic probability for nmCRPC patients. In this study, we developed and externally validated a machine-learning model capable of calculating risk scores and predicting the likelihood of metastasis in nmCRPC patients.

**Patients and methods:**

A total of 2716 nmCRPC patients were included in this study. The training and testing datasets were derived from SPARTAN (NCT01946204) and ARAMIS (NCT02200614), respectively. Regarding metastasis-free survival as the endpoint, we subjected 13 clinical features to 10 machine-learning models and their combinations to predict metastasis. Model performance was assessed through accuracy (AUC), calibration (slope and intercept), and clinical utility (DCA). The risk score calculated by the model and risk factors based on eight identified variates were used for metastatic risk stratification.

**Results:**

The final prognostic model included eight prognostic factors, including novel hormone therapy application, Gleason score, previous treatments received (both surgery and radiotherapy, or neither), Race (White), PSA doubling time (PSADT), hemoglobin (HGB), and lgPSA. The prognostic model resulted in a C-index of 0.724 (95% CI 0.700–0.747) in internal validation and relatively good performance through tAUC (>0.70 at 3-month intervals between 6 and 39 months) in external validation. In the risk score stratifying strategy, compared with the low-risk group, the metastasis HRs for medium- and high-risk groups were 1.72 (95% CI 1.39–2.12) and 4.43 (95% CI 3.66–5.38); as for risk factor count, the HRs are 1.98 (95% CI 1.50–2.61) and 4.17 (95% CI 3.16–5.52), respectively.

**Conclusions:**

In this study, we developed and validated a machine learning prognostic model to predict the risk of metastasis in nmCRPC patients. This model can assist in the risk stratification of nmCRPC patients, guide follow-up strategies, and aid in selecting personalized treatment intensities.

**HIGHLIGHTS**

- This study introduces a machine learning-based model to predict metastasis risk in non-metastatic castration-resistant prostate cancer (nmCRPC) patients, addressing a critical need in the field.
- The model achieved a time-dependent area under the curve greater than 0.7 at 3-month intervals between 6 and 36 months during external validation, demonstrating its robustness and reliability.
- The model allows for effective risk stratification by assigning risk scores or counting risk factors, significantly improving personalized treatment planning and clinical decision-making for nmCRPC patients.

## Introduction

Nonmetastatic castration-resistant prostate cancer (nmCRPC) is characterized by increasing levels of PSA despite androgen deprivation therapy (ADT), without evidence of metastasis on conventional imaging modalities such as computed tomography or bone scintigraphy^[^1^]^. As an advanced stage of prostate cancer, the annual incidence of nmCRPC in the United States was estimated to be approximately 60 000 cases in 2020, with a 34% annual progression rate to metastatic disease and a 16% yearly overall mortality^[^2^]^. Most prostate cancer-related deaths are attributed to widespread metastasis, and a recent study has validated that metastasis-free survival (MFS) can serve as a surrogate endpoint for overall survival (OS) in localized CRPC patients^[^3,4^]^. Therefore, it is necessary to accurately identify nmCRPC patients at high risk of developing metastasis and estimate the time to progression to mCRPC to establish realistic patient expectations and enable precise implementation of treatment and follow-up by healthcare providers.

Currently, the prognosis stratification of nmCRPC primarily relies on the PSA level and the change rate, as reported by PSADT^[^5^]^. Evidence suggests that a shorter PSADT indicates a higher risk of progression^[^6,7^]^, and a PSADT <6 months is sometimes suggested as a cutoff to identify the need for more aggressive therapy^[^8^]^. However, relying on a single binary indicator can only roughly categorize nmCRPC into high-risk and low-risk groups, making it challenging to provide individualized assessments of MFS for patients, limiting its guidance on patient prognosis. In contrast to the lack of well-established prognostic models for nmCRPC patients, high-quality clinical multivariable prognostic models have been established for metastatic castration-resistant prostate cancer (mCRPC) patients, both for chemotherapy-naïve mCRPC and post-chemotherapy mCRPC^[^9,10^]^. In 2023, Halabi *et al* externally validated a previously established mCRPC prognostic model based on the CALGB 90401 Phase III clinical trial (*n* = 1050), with a tAUC (time-dependent area under the curve) of 0.74 (95% CI, 0.73–0.75) in the validation dataset^[^11^]^. Given the current lack of effective and externally validated prognostic models for nmCRPC patients, we aim to leverage data from the latest Phase III clinical trials (SPARTAN and ARAMIS) to identify prognostic and predictive variables for the prognosis of nmCRPC patients and establish a prognostic model tailored explicitly to nmCRPC patients.

In this study, we utilized machine learning methodologies to develop a prognostic model using MFS as the primary endpoint for nmCRPC patients. Considering that novel hormone therapy (NHT) such as enzalutamide, apalutamide, and darolutamide have become the first-line treatment for nmCRPC^[^12,13^]^, we incorporated the prognostic value of NHT application and other clinical data, including baseline PSA level, PSADT, previous treatments received, Gleason score, race, and laboratory indicators, which are related to disease progression, as model parameters. The objective of this study was to create and externally validate a model that calculates risk scores and predicts the probability of metastasis in nmCRPC patients, providing urologists with a valuable tool for clinical practice.

## Methods

### Derivation and validation cohorts

The training dataset was derived from the SPARTAN clinical trial (NCT01946204)^[^14^]^, while the validation dataset was obtained from the ARAMIS clinical trial (NCT02200614)^[^15^]^. These trials shared similar study designs, as well as inclusion and exclusion criteria. ARAMIS enrolled 1509 nmCRPC patients (955 randomized to darolutamide; 554 to placebo), and SPARTAN enrolled 1207 patients (806 randomized to apalutamide; 401 to placebo).

Both ARAMIS and SPARTAN studies were conducted by the Declaration of Helsinki, the International Conference on Harmonisation for Good Clinical Practice, and local regulations^[^16-18^]^. The study protocols were approved by the respective authorities and ethics committees, and signed informed consent was obtained from all patients. Informed consent for constructing and validating the nmCRPC model was optimal due to the de-identified nature of the individual patient data from ARAMIS and SPARTAN. The patient-level data from the SPARTAN clinical trial were obtained through the Yale University Open Data Access Project, while the ARAMIS data were provided by Bayer and made available through Vivli, Inc. The present work has been reported in compliance with REporting recommendations for tumor MARKer prognostic studies (REMARK) criteria and guidelines^[^19^]^.

### Study population

Our study initially included all patients from two clinical cohorts and applied the following criteria for exclusion. Exclusion criteria are as follows: (1) lack of the endpoint result MFS; (2) presence of baseline data missing. Forty-seven patients from SPARTAN and 127 patients from ARAMIS were excluded. Finally, 1160 nmCRPC patients from the Clinical Trial A database and 1247 patients were included in the analysis.

### Study outcome and candidate predictor variables

The study’s outcome was MFS, defined as the time from randomization to evidence of metastasis or death from any cause in both SPARTAN and ARAMIS. A comprehensive literature review identified Candidate predictor variables for potential inclusion in our models. In the data set, 13 variables (NHT application, baseline PSA level, PSADT, previous treatments received, Gleason score, race, and laboratory indicators) were available for analysis. The basic clinical information of the included patients is shown in Table 1.

**Table 1:** Baseline characteristics of patients in the development and validation cohort

Variable Treatment arm	Development cohort (SPARTAN; *n* = 1160)	Validation cohort (ARAMIS; *n* = 1247)
Apalutamide or Darolutamide	776(66.9%)	799 (64.1%)
Placebo	384(33.1%)	448 (35.9%)
N stage		
N0	969(83.5%)	617(49.5%)
N1	191(16.5%)	206(16.5%)
NX	/	424(34.0%)
Bone-sparing agent		
N	1044(90.0%)	1192(95.6%)
Y	116(9.98%)	55(4.41%)
ECOG		
0	883(76.1%)	866(69.4%)
1	227(23.9%)	381(30.6%)
Gleason grade		
<8	654(56.4%)	734(58.9%)
≥8	506(43.6%)	513(41.1%)
Therapy		
Both	349(30.1%)	50(4.01%)
Neither	315(27.2%)	587(47.1%)
Radiotherapy	368(31.7%)	563(45.1%)
Surgery	128(11.0%)	47(3.77%)
Race		
White	770(66.4%)	1016(81.5%)
Asian	129(11.1%)	172(13.8%)
Black	66(5.69%)	47(3.77%)
Others	195(16.8%)	12(0.96%)
Age		
<65	357(30.8%)	168(13.5%)
65–74	508(43.8%)	480(38.5%)
≥75	295(25.4%)	599(48.0%)
PSA double time (months)	4.73 (2.29)	4.93(2.33)
HGB (g/dL)	13.3 (1.22)	13.1(1.19)
ALP (U/L)	80.5(25.1)	79.7(24.0)
ALB (g/dL)	4.37 (0.25)	4.26(0.29)
lgPSA	0.80(0.44)	0.96(0.42)

Binary variables included: NHT application (NHT drugs Darolutamide or Apalutamide or placebo), N stage (N0 or N1), bone-sparing agent (yes or no), ECOG (0 or 1), Gleason grade (<8 or ≥8). Multi-class variables included previous treatments (surgery, radiotherapy, neither, or both applications), race (White, Asian, Black, or Other), and age (<65, 65–74, or ≥74). The remaining parameters were included as continuous variables. To prevent the parameter estimation from being influenced by individual extreme outliers, we performed the subsequent analysis using the log10-transformed PSA values.

To conform to the input format required by all models, multi-class variables are converted into binary variables. Variable selection in the training set was performed using Univariate Cox proportional hazards models. Categorical variables with a *P* value of <0.1 in the univariate analysis were included in the subsequent model building. Additionally, split multi-class variables, with any of the binary variables’ *P* value <0.1, were included in the following procedure.

### Model derivation

After data analysis variable selection, we preliminarily assessed the performance of 10 machine learning models allowing the use of survival data [Least absolute shrinkage and selection operator (LASSO), stepwise Cox proportional hazards (Stepwise Cox), partial least squares proportional hazard regression (plsRCox), CoxBoost, Efficient Neural Network (Enet), gradient boosting machine (GBM), random survival forest (RSF), Survival Support Vector Analysis (survivalSVM), and Supervised Principal Components (superPC)] and their combinations. Further details regarding the machine learning methods have been published previously^[^20^]^.

**Figure 1.**
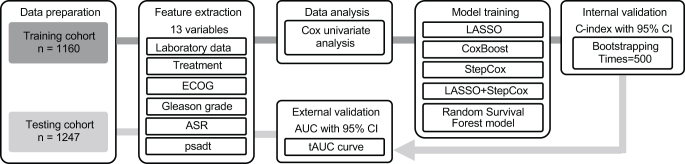
The framework of machine-learning model development for MFS prediction.

After comprehensively evaluating all the machine learning models, we selected five models with centered and scaled continuous variables due to their varying scales. These models include a Stepwise Cox model^[^21^]^, a LASSO model^[^22^]^, a LASSO and Stepwise Cox combined model^[^23^]^, a CoxBoost model^[^24^]^, and an RSF model^[^25^]^. The overall framework of machine-learning model development is illustrated in Fig. 1.

### Prediction model performance

To assess the accuracy of these five models, we evaluated the model performance through internal and external validation. C-indexes and 95% CI were calculated using bootstrapping (500 times) to evaluate the models’ accuracy. In the validation cohort, predictive accuracy was acquired by tAUC assessed by plotting the time-AUC curve at 3-month intervals between 6 and 36 months. The hazard ratio (HR) and 95% confidence interval (CI) were computed for each potential predictor. In addition to accuracy, it is recommended to assess calibration and clinical utility in developing a prognostic model. Therefore, we employed calibration curves and decision curve analysis to evaluate these aspects in the training and testing cohorts. Multicollinearity was calculated using Spearman correlation and variance inflation factor (VIF).

To further support the clinical use of the model, a risk score was developed from the multivariable model in both the training and testing sets. We stratified the patients into three risk groups according to the risk score trisection value calculated in the training set.

Moreover, we built another three-risk prognostic group based on the number of risk factors converted from model predictors. Table S1 (available at: http://links.lww.com/JS9/E13) defines these risk factors. To achieve a balanced distribution, patients were stratified into low-risk (0-2 risk factors), medium-risk (3-4 risk factors), or high-risk (>4 risk factors) groups.

The Kaplan–Meier curve was applied to estimate MFS and OS distribution by prognostic risk groups with and without placebo patients, and the log-rank statistic was applied to assess the differences in MFS among three-risk groups. Median MFS, metastasis-free survival probability, and hazard ratio with 95% CI were calculated for the three risk groups. The statistical analysis was performed using R Studio, version 4.0.2. Statistical significance was defined as a two-sided *P* value of less than 0.05 (*P* < 0.05).

## Results

### Characteristics of the cohorts

A total of 1160 participants were included in the derivation cohort (SPARTAN) and 1247 in the external validation cohort (ARAMIS). Based on our exclusion criteria, 47 patients from the full SPARTAN population (*n* = 1207) and 262 patients from the full ARAMIS population (*n* = 1509) were excluded from the analysis. The baseline characteristics of patients in the derivation and validation cohort are shown in Table 1. Dating to the cutoff date, the MFS times distribution of the development and validation cohorts were presented in Figure S1 (available at: http://links.lww.com/JS9/E13).

### Prognostic factors influencing MFS

Before conducting the univariate analysis, we converted multi-class variables (such as previous therapy received, race, and age) into binary variables to facilitate the following model-building step. We used the univariate Cox proportional hazards model to analyze the relationship between the following parameters and MFS (Table 2): treatment, N stage, Bone-sparing agent use, ECOG score, Gleason score, previous therapy (surgery, radiotherapy, both, or neither), race (White, Asian, Black, and others), age (<65, 65–74, and ≥75), PSADT, HGB, alkaline phosphatase (ALP), albumin (ALB), and lgPSA. Among the parameters associated with MFS, NHT, PSADT, and lgPSA application exhibited strong correlations with MFS prognosis, previously reported as OS risk factors in nmCRPC patients^[^26^]^.

**Table 2:** Univariate analysis of patients’ characteristics and MFS in the development cohort

Variable	HR	95% CI	*P* value
Treatment arm^a^	0.276	(0.230, 0.332)	<0.001
N stage	1.197	(0.945, 1.515)	0.136
Bone-sparing agent	1.005	(0.748, 1.350)	0.975
ECOG	1.282	(1.046, 1.572)	0.017
Gleason grade^b^	1.203	(1.004, 1.440)	0.045
Therapy			
Both	1.223	(1.011, 1.479)	0.038
Surgery	0.876	(0.650, 1.180)	0.383
Radiotherapy	1.123	(0.930, 1.355)	0.229
Neither	0.737	(0.593, 0.916)	0.006
Race			
White	1.229	(1.012, 1.494)	0.038
Asian	0.850	(0.612, 1.181)	0.333
Black	0.932	(0.628, 1.384)	0.726
Others	0.824	(0.648, 1.048)	0.115
Age			
<65	0.959	(0.790, 1.164)	0.672
65–74	0.996	(0.831, 1.194)	0.967
≥75	1.055	(0.858, 1.298)	0.610
PSA double time	0.881	(0.845, 0.918)	<0.001
HGB	0.919	(0.852, 0.991)	0.028
ALP	1.002	(0.998, 1.005)	0.345
ALB	0.898	(0.631, 1.278)	0.551
lgPSA	1.593	(1.302, 1.949)	<0.001

^a^ NHT (apalutamide) vs. placebo.

^b^ Gleason grade ≥8 vs. Gleason grade <8.

It was noted that the common prognostic parameter lactate dehydrogenase (LDH) was not included due to a high number of missing values. The categorical variables (N stage, bone-sparing agent use, and age) with *P* value >0.1 were excluded in the following steps, while all the continuous variables were preserved. Additionally, we observed that white people had an HR of 1.229 (95% CI: 1.012–1.494; *P* = 0.038). In order to make this model applicable to all ethnicities, the race variable was preserved.

### Selection of prognostic models

After screening the predictors through univariate analysis, we fitted 101 machine learning models in the training cohort. We calculated the AUC between training and testing cohorts using the method published by the previous study^[^20^]^. As shown in Table S2 (available at: http://links.lww.com/JS9/E13), we chose the RSF model because its mean AUC was superior to the other models, while a significant gap existed between the training and testing cohort attributed to the overfitting probability. Subsequently, we selected four other representative models, including the Stepwise Cox model, the LASSO, the LASSO + Stepwise Cox combined model, and the CoxBoost model. They presented relatively excellent predictive efficiency (AUC>0.7), performance consistency (the AUC discrepancy between two cohorts), model simplicity (fewer variables), as well as wide usage in prognostic model development (high level of acceptance and replication convenience).

**Figure 2.**
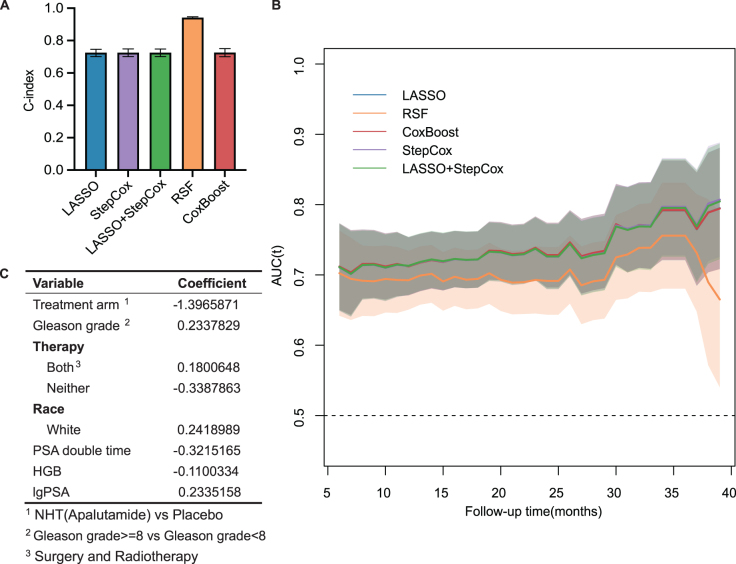
Internal and external evaluation of machine-learning models. (A) C-indices of models calculated by bootstrapping (times = 500) in the training cohort. (B) Time-dependent AUC curve of models in the testing cohort at 3-month intervals between 6 and 39 months. (C) The parameters and coefficients of the LASSO + Stepwise Cox model.

This study recommended using all available data in the dataset to develop the model and estimate its performance^[^27^]^. Thus we took the bootstrapping resampling approach instead of randomly splitting the training cohort to acquire C-index and 95% CI in five models. Random forest model exhibited highest C-index 0.941 (95% CI 0.934–0.947), while other models’ C-index were approximately the same [C-index with 95% CI: LASSO 0.724 (0.701–0.750), CoxBoost 0.725 (0.700–0.749), LASSO + Stepwise Cox 0.724(0.700–0.747), and Stepwise Cox 0.725 (0.702–0.749)] (Fig. 2A).

**Figure 3.**
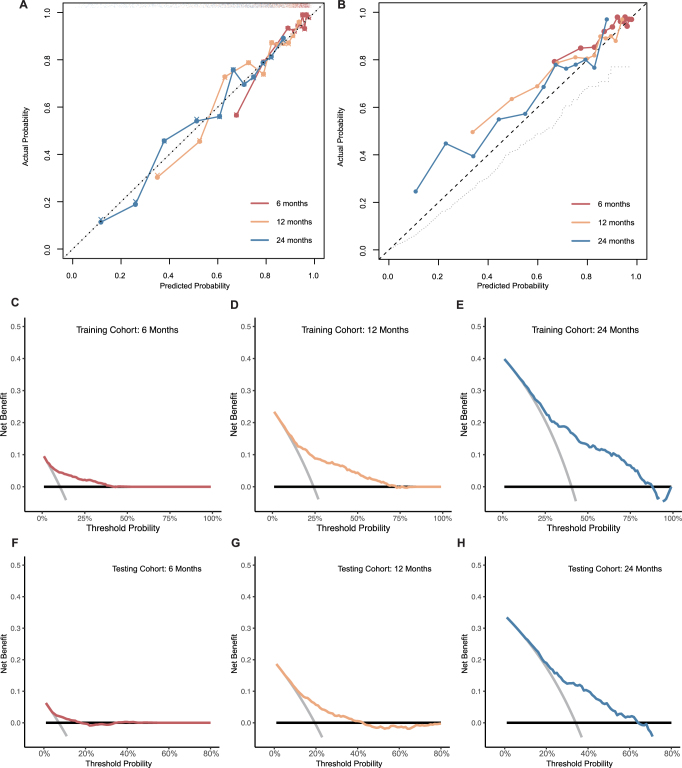
(A–B) Calibration curves of the training and testing cohort, respectively, in 6, 12, and 24 months. (C–H) Decision curve analysis of the training and testing cohort in 6, 12, and 24 months.

In the following, we further evaluated the performance of these models in the external dataset ARAMIS. Despite the excellent performance in internal validation, the RSF model showed significantly and consistently poor performance compared to the other four models. In all five models constructed, the LASSO + Stepwise Cox model presented the highest prediction accuracy (Fig. 2B). And the number of parameters (eight) chosen by these models is minimum (Table S2, available at: http://links.lww.com/JS9/E13). Given the model’s simplicity and broad applicability, the LASSO + Stepwise Cox model was selected as the optimal prognostic model for subsequent analysis. The detailed model establishment procedure (LASSO for variable screening, multivariate Cox model analysis, multicollinearity measurement through Spearman correlation and VIF) was presented in Figure S2 (available at: http://links.lww.com/JS9/E13). Meanwhile, the model presented excellent calibration ability in two cohorts because their calibration curves were close to the diagonal line (Fig. 3A and B). Furthermore, we adopted decision curve analysis to assess our model’s clinical utility. In the training cohort, DCA curves (Decision Curve Analysis curves) showed that if the threshold probability of patients is 5–40%, 10–74%, and 12–89%, using nomogram in the current study to predict metastasis risk respectively in 6, 12, and 24 months could add more benefit (Fig. 3C–E). As for the testing cohort, the DCA curves illustrated that the threshold probability is 4–18%, 10–42%, and 12–63%, corresponding to 6, 12, and 24 months (Fig. 3F–H).

### Clinical utility of the prediction model

#### Risk groups based on the risk score

**Figure 4.**
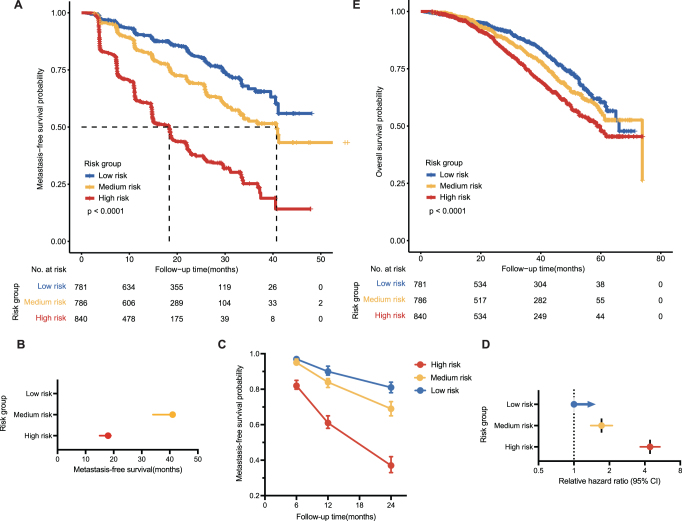
Risk groups based on the risk score. (A) Kaplan–Meier survival curves for MFS of the three-risk groups in the training combined validation sets. (B–D) Mean MFS times, nonmetastasis probability, and relative hazard ratio with 95% CI for the three-risk groups. (E) Kaplan–Meier survival curves for OS of the three risk groups in the training combined validation sets.

The risk score acquired from the model can serve as the basis for metastatic risk stratification in clinical practice or for the randomization of patients in clinical trials. For three-risk groups, through getting cutoff value using the training data, there were respectively 781 patients (32.4%), 786 patients (32.6%), and 840 patients (34.9%) in the low-, medium-, and high-risk groups with unreached median MFS, median MFS times of 41 months (95% CI 34 months, not reached) and 18 months (95% CI 15–19, log-rank test *P* < 1 × 10^−4^; Fig 4A and B). The nonmetastasis probability decreases over time, with the high- and medium-risk group substantially higher than the low-risk group in 12, 34, and 36 months (Fig. 4C). Compared with the low-risk group, the metastasis HR for medium- and high-risk groups were 1.72 (95% CI 1.39–2.12) and 4.43 (95% CI 3.66–5.38; Fig 4D), respectively. Meanwhile, the risk stratification also presented a significant overall survival (OS) difference, enhancing its clinical utility (Fig. 4E).

#### Risk groups based on the risk factor number

To facilitate rapid assessment of patients’ metastasis probability and address patient inquiries, parameters in the model were converted into risk factors according to Table S1 (available at: http://links.lww.com/JS9/E13). Thus, even without a risk score obtained from the model, physicians can make an initial assessment of patients’ condition by calculating the number of risk factors.

**Figure 5.**
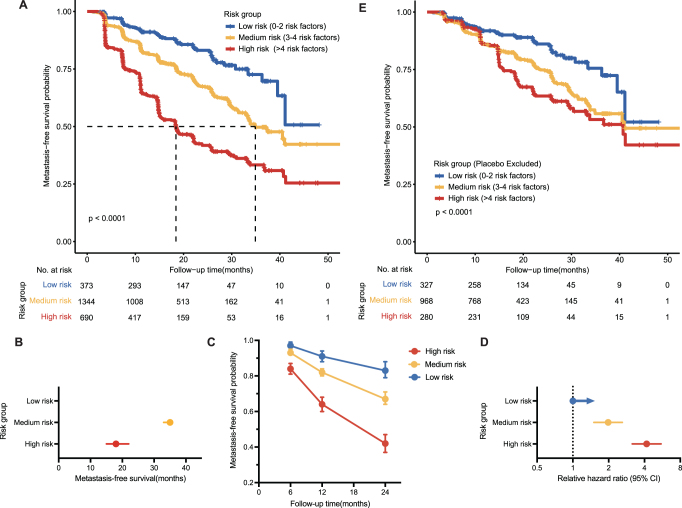
Risk groups based on the risk factors. (A) Kaplan–Meier survival curves for the three-risk groups in the training combined validation sets. (B–D) Mean MFS times, nonmetastasis probability, and relative hazard ratio with 95% CI for the three-risk groups. (E) Kaplan–Meier survival curves for MFS of the three risk groups excluded placebo patients in the training combined validation sets.

Divided into three-risk grouping in training combined validation datasets, respectively, 15.5%, 55.8%, and 28.7% of patients were in the low- (0–2 risk factors), medium- (3–4 risk factors), and high-risk (>4 risk factors) groups. The median MFS was not reached in the low-risk group, 35 months in the medium-risk group, and 18 months in the high-risk group (Fig. 5A and B). In months 12, 34, and 36, the high- and medium-risk groups showed higher nonmetastasis probabilities than the low-risk groups (Fig. 5C). Compared with the low-risk group, the metastasis HRs for the medium- and high-risk groups were 1.98 (95% CI 1.50–2.61) and 4.17 (95% CI 3.16–5.52; Fig 5D), respectively. By contrast, metastasis risk stratification based on risk count presented accuracy similar to that of risk number. In order to conform to new therapy guidelines approved by the FDA, we excluded placebo patients, and NHT patients still exhibited prognostic discrepancy according to division by the risk number (Fig. 5E).

## Discussion

In this study, we developed a prognostic model and risk stratification for metastasis based on two latest independent Phase III clinical trials’ cohorts, which complements the blank of the multivariate prognostic model for MFS and OS in nmCRPC patients. 10 algorithms were utilized to construct 101 kinds of machine learning models for MFS, and LASSO + Stepwise Cox model presented several advantages over other models such as a better predictive ability and simplicity. The performance of the selected prognostic model was comprehensively evaluated from three perspectives: accuracy (AUC in internal and external validation), calibration (calibration curve), and clinical utility (DCA). By risk score and variants acquired by the LASSO + Stepwise Cox model, we built two risk stratification strategies to address clinical requirements for disease stage assessment and individual therapy scheme design in nmCPRC patients.

Prior studies have focused on exploring single risk factors for MFS^[^28-32^]^, but a multivariate prognostic model is lacking to evaluate and stratify individuals’ metastatic risks. The chosen model identified eight prognostic predictors of MFS: treatment, Gleason score, previous therapy (both surgery and radiotherapy, or neither), Race, PSADT, HGB, and lgPSA. It included the widely accepted factors with prognostic value in prostate cancer patients such as NHT application^[^14,15^]^, Gleason score^[^33^]^, PSADT^[^5^]^, and PSA^[^29,34^]^. In addition, we have identified several new prognostic factors for MFS: previous therapy (surgery, radiotherapy, or both), race, and HGB levels. We found that patients who had previously undergone local surgery and radiotherapy had a higher risk of distant metastasis. This may be attributed to the selective pressure exerted on tumor cells by local treatment, leading to a higher malignancy and increased metastatic potential in cells that recurred after both surgery and radiotherapy. Moreover, the analysis revealed that being of White race became a significant risk factor in this model (HR 1.27 with 95% CI 1.04–1.55). For ethnicity impact on prognosis, the earlier finding showed white men had a better prognosis than black men^[^35^]^. However, recent studies have observed the prognostic discrepancy lies in the differing access to medical settings. Specifically, after adjusting for confounding factors, American black patients were found to have a reduced risk of metastatic disease or death compared to white patients^[^36^]^. Our previous study proposed that Asians with de novo metastatic prostate cancer have a better prognosis than White^[^37^]^. However, direct evidence supporting similar conclusions in nmCRPC is lacking. Thirdly, HGB levels were identified as a risk factor across various stages of prostate cancer^[^38,39^]^, indicating a relationship with the response and tolerance capacity to prior therapy. It is reasonable to deduce that nmCRPC patients with decreasing HGB levels have a lower response to treatment, resulting in high metastatic probability.

Our model focused on pretreatment survival prediction and did not incorporate post-treatment prostate-specific antigen (PSA) kinetics or radiological features, which have been shown to have a significant correlation with prognosis^[^28,40,41^]^. Utilizing post-treatment data resulted in the exclusion of patients who experienced metastasis or dropped out of the clinical trial within 3 and 6 months, causing available data loss and population bias toward the NHT group. In contrast, our pretreatment model provides more comprehensive coverage for nmCRPC patients. By considering therapy tolerance in conjunction with the primary cancer condition, our model significantly facilitates clinical decision-making and enhances its practical application. The estimation and stratification of prognosis can assist clinicians in determining the appropriate aggressiveness of therapy. This enables individualized treatment approaches to be implemented in the context of clinical trials, with more aggressive strategies and combination therapies for high-risk patients while offering alternative medical schemes with fewer side effects and improved quality of life for low-risk patients. This can also help explore combination treatment strategies for high-risk individuals in future research, such as the ongoing clinical trial NCT05849298. Additionally, the value of local therapies in controlling the progression of high-risk nmCRPC should also be considered.

Our research has some limitations. First, the exclusion of data from recent trials is inevitable due to missing value, leading to a reduction in the available dataset and may have introduced a potential selection bias. Secondly, although the variables included have clinical rationale and statistical significance in univariate analysis, outcomes for individuals in clinical practice may exist differently from the populations from clinical trials. Both the SPARTAN and ARAMIS trials predominantly enrolled patients with PSA doubling time <10 months, which limits the generalizability of our model to lower-risk populations, such as those with PSA doubling time >10 months or Gleason 3 + 3 disease. Therefore, external validation should be conducted in independent cohorts with diverse settings, particularly in real-world contexts, to assess the broader applicability of the model.

Additionally, it is essential to note that with the advancement of novel molecular imaging techniques, nearly half of the nmCRPC patients can now be identified with PSMA-positive metastases^[^42^]^. However, this does not diminish the utility of our model. First, patients with PSMA-positive metastases and conventional imaging-negative nmCRPC may exhibit biological differences compared to conventional imaging-detected mCRPC. According to Sutera *et al*^[^43^]^, patients with oligoM CSPC detected by advanced molecular imaging had a lower incidence of TP53 mutation (17% vs. 28%) than those detected by conventional imaging. Therefore, the direct incorporation of PSMA for guiding treatment decisions in nmCRPC is controversial, as current disease classification, treatment, and outcome assessment are based on conventional imaging. For instance, it remains unclear whether the improved outcomes observed in PSMA-defined oligoM CRPC (conventional imaging-negative nmCRPC) are due to a lower tumor burden or indolent biology. Furthermore, it would be inappropriate to classify this subset of PSMA-PET positive but conventional imaging-negative patients directly as mCRPC. Despite having lesions detected by novel molecular imaging, their prognosis is significantly better than that of mCRPC patients defined by conventional imaging according to current guidelines. For instance, in ARAMIS, the median overall survival (OS) for nmCRPC patients treated with darolutamide was 40.4 months, and in SPARTAN, the median MFS for nmCRPC patients treated with apalutamide was 40.5 months. In contrast, the median OS in existing prognostic models for mCRPC patients is only 32.7 months^[^10^]^. Therefore, the subgroup of CRPC patients who are negative on conventional imaging but positive on PSMA-PET represents a distinct patient population with unique prognostic characteristics.

Second, PSMA PET, as a novel imaging modality, lacks high-level evidence supporting its role in the prognostic stratification of nmCRPC patients. Only retrospective studies, such as the one conducted by Manuel *et al*^[^42^]^, have been performed, demonstrating that only nmCRPC patients with more than five metastatic lesions detected by PSMA PET imaging showed a statistically significant long-term survival disadvantage. Furthermore, due to the cohort being constructed in the pre-NHA era (2013–2018), the treatment approaches in the cohort were diverse. Some patients received local or regional therapies, while others underwent stereotactic body radiation therapy for metastatic lesions. Additionally, most patients did not receive NHA treatment when transitioning to the CRPC stage. Considering these confounding treatment factors, the prognostic value of PSMA PET in nmCRPC patients remains uncertain. Future prospective studies are necessary to evaluate the prognostic advantage or alteration conferred by PSMA PET detection. Therefore, there is still a long way to go before new modalities can reclassify nmCRPC patients, and during this period, our model can demonstrate its clinical utility.

Third, our model utilizes data from robust phase 3 trials, incorporating comprehensive and reliable demographic parameters, clinical indicators, and pathological parameters, with less selection bias and a large number of patients, thereby providing strength in model performance. The external validation results prompt a quick translation into clinical practice. In contrast, no current phase 3 nmCRPC trial incorporates PSMA PET, significantly impacting the evidence’s strength. Lastly, the user-friendliness of our model provides convenience and cost-saving in clinical usage, trial planning, and patient consultation, especially considering that PSMA PET is not reimbursed for nmCRPC in many countries.

In conclusion, we have successfully constructed and validated a prognostic model of eight variables for patients with nmCRPC. These prognostic factors can calculate a risk score, serving as an eligibility criterion and aiding in stratified randomization for clinical trials. Additionally, the risk score can help identify subsets of patients with significantly different outcomes regarding metastasis-free survival, allowing for swift identification of high-risk individuals who may require more aggressive treatment approaches.

## Data Availability

The data that support the findings of this study are available from YODA (https://yoda.yale.edu/applications) and Vivli (https://search.vivli.org/), but restrictions apply to the availability of these data, which were used under license for the current study. Data are however available from the authors upon reasonable request and with permission of YODA and Vivli.

## Research registration unique identifying number (UIN)

SPARTAN (NCT01946204); ARAMIS (NCT02200614).
